# Evaluation of Sample Preparation Methods for Fast Proteotyping of Microorganisms by Tandem Mass Spectrometry

**DOI:** 10.3389/fmicb.2019.01985

**Published:** 2019-09-06

**Authors:** Karim Hayoun, Duarte Gouveia, Lucia Grenga, Olivier Pible, Jean Armengaud, Béatrice Alpha-Bazin

**Affiliations:** Laboratoire Innovations Technologiques pour la Détection et le Diagnostic, Service de Pharmacologie et Immunoanalyse, CEA, INRA, Bagnols-sur-Cèze, France

**Keywords:** detection, identification, mass spectrometry, proteotyping, sample preparation, shotgun proteomics, microorganisms

## Abstract

Tandem mass spectrometry-based proteotyping allows characterizing microorganisms in terms of taxonomy and is becoming an important tool for investigating microbial diversity from several ecosystems. Fast and automatable sample preparation for obtaining peptide pools amenable to tandem mass spectrometry is necessary for enabling proteotyping as a high-throughput method. First, the protocol to increase the yield of lysis of several representative bacterial and eukaryotic microorganisms was optimized by using a long and drastic bead-beating setting with 0.1 mm silica beads, 0.1 and 0.5 mm glass beads, in presence of detergents. Then, three different methods to obtain greater digestion yield from these extracts were tested and optimized for improve efficiency and reduce application time: denaturing electrophoresis of proteins and in-gel proteolysis, suspension-trapping filter-based approach (S-Trap) and, solid-phase-enhanced sample preparation named SP3. The latter method outperforms the other two in terms of speed and delivers also more peptides and proteins than with the in-gel proteolysis (2.2 fold for both) and S-trap approaches (1.3 and 1.2 fold, respectively). Thus, SP3 directly improves tandem mass spectrometry proteotyping.

## Introduction

Taxonomical identification of microorganisms has been considerably simplified by the use of mass spectrometry. Proteotyping by MALDI-TOF mass spectrometry relies on recording the mass profiles of small molecular weight and basic polypeptides released from the sample and comparing them with those recorded previously for thousands of microorganisms under similar conditions (Lavigne et al., 2013; Grenga et al., 2019). This approach is simple, rapid and low cost, but requires first to isolate each microorganism on an agar plate. Proteotyping allows characterizing microorganisms in terms of taxonomy with a high specificity as even subspecies can be discriminated (Durighello et al., 2014). Recently, proteotyping by tandem mass spectrometry was applied for complex samples (Karlsson et al., 2015, 2018; Berendsen et al., 2017; Kleiner, 2019). In this case, proteotyping consists in assigning protein or peptide sequences analyzed by mass spectrometry to a taxonomical database for taxonomical identification of microorganisms. In metaproteomics, peptides are identified after comparing experimental spectra with a protein sequence database and then the peptide sequences are analyzed in terms of taxonomical representation and specificity by means of the last common ancestor search (Mesuere et al., 2015). On this principle, microorganisms can be identified from complex samples and their respective biomass contributions can be established (Kleiner, 2019). Beside taxonomical identification, functional determinants can be obtained such as peptide signature of several antibiotic resistances (Trip et al., 2015). Two tandem mass spectrometry strategies can be implemented for this: (i) shotgun proteomics where information is recorded for identifying a large panel of peptides (Boulund et al., 2017) and (ii) targeted proteomics using selected reaction monitoring for certifying the presence of a specific set of discriminant peptides (Charretier et al., 2015; Cecchini et al., 2018). Compared to MALDI-TOF mass spectrometry, tandem mass spectrometry coupled to liquid-chromatography (LC-MS/MS) establishes many more molecular sequence determinants and is thus more powerful for discriminating microorganisms. This approach was even shown to be able to discriminate spores and vegetative cells of *Bacillus atrophaeus* (Mappa et al., 2018).

Advances in tandem mass spectrometry in the last decade allow a deeper characterization of microorganisms (Armengaud, 2013, 2016). For this, proteins should be extracted and purified after cell lysis, and then digested into peptides using an endoprotease, classically trypsin. The resulting peptides are resolved using LC-MS/MS. The recorded MS/MS spectra are then interpreted using a protein sequence database derived from the annotated genomes of the organisms present in the sample. In this approach, the numbers of peptides and proteins that are identified are largely dependent on the protein extraction yield and on the proteolysis efficiency (Tanca et al., 2013; Tubaon et al., 2017). Thus, sample preparation is a critical step for tandem mass spectrometry proteotyping.

An efficient protein extraction requires a performant cell disruption method within an appropriate protein solubilization buffer. The nature of the biological samples and type of microorganisms may strongly influence the yield of lysis and protein extraction. Among the different lysis methods, bead beating was reported to be successful for a large diversity of samples (Tanca et al., 2014; Warnke et al., 2016; Zhang et al., 2018). Tanca et al. (2014) demonstrated that bead-beating allows a better extraction yield in comparison to heating in detergent-based buffer for gram-negative bacteria, gram-positive bacteria and yeasts. Swarge et al. (2018) have shown that bead-beating is adapted for extraction of proteins from sporulated cells. Lysis is performed in presence of detergents such as sodium dodecyl sulfate (SDS) for the solubilization of cellular and hydrophobic membrane proteins (Tanca et al., 2013; Weston et al., 2013; Zhang et al., 2018). Different strategies have been proposed to remove the detergent inhibiting the trypsin activity and other possible contaminants that may disturb proteolysis and/or subsequent LC-MS/MS analysis. Among them, the most commonly used method is subjecting proteins to electrophoresis in denaturing conditions on a polyacrylamide gel prior to in-gel digestion (Hartmann et al., 2014; Kim and Cho, 2019). Protein precipitation is another well-known method to concentrate the components of interest, but their difficult subsequent solubilization may cause material loss (Eddhif et al., 2018). Support-aided methods, such as suspension trapping [S-Trap (Zougman et al., 2014)] and single-pot solid-phase-enhanced sample preparation [SP3 (Hughes et al., 2014)], are interesting alternatives and are gaining momentum. S-Trap is a new powerful Filter-Aided Sample Preparation (FASP) method that consists in trapping acid aggregated proteins in a quartz filter prior enzymatic proteolysis. The generated peptides are eluted and then concentrated for LC-MS/MS analysis. In comparison to classical FASP approach (Wisniewski et al., 2009; Wisniewski, 2017), S-Trap method presents the advantage to decrease handling steps resulting in shorter sample preparation. For the SP3 method proteins are captured on the surface of carboxylate-functionalized magnetic beads. The use of a magnetic rack simplifies the washing steps and the recovery of peptides.

In this study, we first optimized the lysis of a mixture of representative microorganisms including prokaryotes and eukaryotes using beads-beating. Then, we explored the performances of in-gel, S-Trap and SP3 protocols. We also optimized these three protein digestion approaches in order to obtain a fast preparation of peptides for tandem mass spectrometry proteotyping. SP3 delivers a higher coverage of the sample with higher numbers of assigned unique peptide sequences and identified proteins, while being the quickest method.

## Materials and Methods

### Strains

*Bacillus subtilis* ATCC6633 and *Bacillus cereus* ATCC14579 strains were purchased from the American type culture collection (ATCC). *Escherichia coli* BL21(DE3) was obtained from a pET Expression System 30 kit (Novagen) and *Saccharomyces cerevisiae* was the baker’s yeast l’Hirondelle (Lesaffre, France). *Pseudomonas aeruginosa* CIP104116 and *Acinetobacter baumanii* CIP70.10 were obtained from the Collection of Institut Pasteur (CIP).

### Bead-Beating Mixtures

Mixtures of beads used for cell disruption were: (i) “Beads mixture A” (BMA) containing 2/3 silica beads 0.1 mm (MP Biomedicals) and 1/3 glass beads 0.5 mm (Bertin Technologies), (ii) “Beads mixture B” (BMB) containing 2/3 silica beads 0.1 mm and 1/3 glass beads 0.1 mm (Bertin Technologies), and (iii) “Beads mixture C” (BMC) corresponding to 1/3 of each bead types.

### Microbial Cultures

Bacteria were cultured for 16 h under aerobic conditions at 30°C at 140 rpm with the following media: brain heart infusion medium (Biomerieux) for *B. subtilis*, Lysogeny broth (BD Bacto) for *E. coli* and *B. cereus*, and Tryptic Soy Broth (Biomerieux) for *A. baumanii*, *K. aerogenes* and *P. aeruginosa*. *S. cerevisiae* cells (82 mg of baker’s yeast) were diluted in 25 mL of PBS 1X buffer pH 7.4 (Gibco). Aliquots of cells were prepared from 250 μL of cell culture at OD_600__*nm*_ equal to 1, thus corresponding to 1 × 10^7^ colony forming units (CFU) of *B. subtilis*, 1 × 10^8^ CFU of *E. coli*, or 3 × 10^7^ CFU of *S. cerevisiae*. An asymmetric mixture of microorganisms, named “Mix3,” containing 55% of *B. subtilis*, 35% of *E. coli*, and 10% of *S. cerevisiae* cells was prepared to obtain a final quantity of 1 × 10^7^ CFU per tube. Cells were collected by centrifugation at 8,000 × *g* for 5 min and stored at −20°C until use.

### Cell Lysis by Bead Beating, Protein Extraction, and Evaluation of the Yield of Extraction

Cell pellets were treated essentially as previously described (Mappa et al., 2018). First, they were homogenized in lithium dodecyl sulfate (LDS) 1X lysis buffer (60 μL per mg of pellet) containing 26.5 mM Tris/HCl, 35.25 mM Tris base, 0.5% lithium dodecyl sulfate (w/v), 2.5% glycerol (w/v), 0.13 mM EDTA, 0.06 mM SERVA Blue G-250, and 0.04 mM phenol red, buffered pH 8.5, and supplemented with 5% beta-mercaptoethanol (v/v). Samples were incubated for 5 min at 99°C in a thermomixer (Eppendorf) and sonicated 5 min in an ultrasonic water bath (VWR ultrasonic cleaner). Samples were then transferred into 2 mL Screw Cap microtubes (Sarstedt) containing 200 mg of beads. Cell disruption was performed with a Precellys Evolution instrument (Bertin Technologies) operated at 7,800 or 10,000 rpm for 3 or 10 cycles of 30 s, with 30 s of pause between each cycle. After lysis, samples were centrifuged at 16,000 × *g* for 1 min and the resulting supernatant was transferred to a new microcentrifuge tube before being incubated at 99°C for 5 min. For densitometric evaluation of protein extraction yield, a volume of 12.5 μL of sample was loaded on NuPAGE 4–12% Bis-Tris gel for a 3 min electrophoresis migration at 200 V in MES/SDS 1X running buffer. The proteins were stained for 30 min with Coomassie SimplyBlue SafeStain (Thermo Fisher Scientific). Densitometry data values were acquired with a GS-800 Densitometer Calibrator (BioRad) and analyzed with Quantity One 1-D analysis software (BioRad).

### In-Gel Trypsin Proteolysis

For in-gel proteolysis, protein lysates (20 μL) were subjected to short SDS-PAGE migration as described by Hartmann et al. (2014). The resulting polyacrylamide bands containing the whole proteome were sliced and placed in 96 well-plate. A reduction step was performed using 25 mM dithiothreitol in 50 mM NH_4_HCO_3_ at 56°C for 10 min, followed by an alkylation step with 55 mM iodoacetamide in 50 mM NH_4_HCO_3_ for 10 min at room temperature in the dark. Proteolysis was enhanced by gel rehydration with 20 μL of 50 mM NH_4_HCO_3_ containing 0.2 μg of trypsin gold (Promega) supplemented with 0.01% ProteaseMAX surfactant (Promega) during 15 min for trypsin penetration into gel bands. The trypsin overflow was removed before adding 50 μL of 50 mM NH_4_HCO_3_ with 0.01% ProteaseMAX surfactant and incubation at 50°C for 60 min (standard) or 15 min (fast digestion) for proteolysis. The resulting peptide pools extracted from the gel bands were acidified with 0.5% trifluoroacetic acid (TFA) final concentration.

### S-Trap Trypsin Proteolysis

Proteins from cellular lysates were reduced with a solution of 20 mM tris (2-carboxyethyl)phosphine (TCEP) in 50 mM NH_4_HCO_3_ as previously described (Kulak et al., 2014) supplemented with 55 mM iodoacetamide in 50 mM NH_4_HCO_3_, then incubated for 10 min at 56°C followed by 10 min incubation at room temperature in the dark. The proteins were acidified with a final concentration of 1.2% phosphoric acid and diluted with 6 volumes of S-Trap buffer [90% Methanol, 100 mM triethylammonium bicarbonate (TEAB), pH 7.1] to aggregate proteins in colloidal particles. The samples were then transferred into S-Trap Micro Spin columns (Protifi), trapped in filter by two centrifugations at 4,000 × *g* for 1 min, washed twice with 150 μL of S-Trap buffer, and centrifuged at 4,000 × *g* for 1 min. Proteolysis was initiated with the addition into the S-Trap cartridge of 20 μL of 50 mM NH_4_HCO_3_ supplemented with 2 μg of trypsin and 0.01% ProteaseMAX detergent, followed by a 15 min or 60 min incubation at 50°C. Peptide elution was conducted with: (i) 40 μL TEAB 50 mM, (ii) 0.2% formic acid (HCOOH) in H_2_O, and (iii) 35 μL of 50% acetonitrile (CH_3_CN) and 0.2% formic acid final concentration, with centrifugations at 4,000 × *g* for 1 min between each elution buffer. Eluates were pooled, lyophilized in a speed vacuum and re-suspended in 10 μL of 50 mM NH_4_HCO_3_ buffer containing 0.5% TFA.

### SP3 Proteolysis

The reduction and alkylation steps were performed as previously described for the S-Trap trypsin proteolysis. A stock solution of Sera-Mag magnetic carboxylate modified particles was obtained by mixing 500 μg of each hydrophylic and hydrophobic bead type (commercial solution at 10 mg/mL) followed by addition of 100 μL of milli-Q water. Beads were retained with a neodymium magnet N42, with nickel (supermagnete; reference Q-40-20-10-N) and washed twice with 200 μL of milli-Q water. After washing, beads were re-suspended in 100 μL of milli-Q water in order to have a 10 μg per μL stock solution that was stored at 4°C until use. Forty μg of Sera-Mag particles stock solution at 10 μg/μL were added to the sample, and then acidified with half volume of formic acid. Protein binding to magnetic beads was activated with the addition of CH_3_CN at a final concentration of 85%. The protein–bead complex was retained with a neodymium magnet for supernatant removal and was submitted to 3 washing steps of 30 s, twice with 70% ethanol, and 100% CH_3_CN for the last step. Paramagnetic beads were re-suspended in 10 μL of digestion buffer containing 1 μg/μL of trypsin gold in 50 mM NH_4_HCO_3_ and incubated for 15 min or 60 min at 50°C. The resulting peptides were recovered from the beads using the magnet and then acidified with 0.5% TFA final concentration.

### Liquid Chromatography and Tandem Mass Spectrometry

Peptides were identified using an ultimate 3000 nano LC system (Thermo Fisher Scientific) coupled to a Q-Exactive HF mass spectrometer (Thermo Fisher Scientific) as previously described (Klein et al., 2016). Peptides from the Mix3 (0.3 μg) were desalted on a reversed-phase PepMap 100 C18 μ-precolumn (5 μm, 100 Å, 300 μm i.d. × 5 mm, Thermo Fisher Scientific) before peptide separation on a nanoscale PepMap 100 C18 nanoLC column (3 μm, 100 Å, 75 μm i.d. × 50 cm, Thermo Fisher Scientific) at a flow rate of 0.3 μL.min^–1^ using a 60 min gradient of mobile phase A (0.1% HCOOH/100% H_2_O) and phase B (0.1% HCOOH/90% CH_3_CN). The gradient used was: 2.5% B for 0 to 3 min, 2.5–25% B from 3 to 53 min and 25–40% B from 53 to 63 min. The mass spectrometer was operated in Top20 mode. Full MS were acquired from 350 to 1,800 *m/z* and the 20 most abundant precursor ions were selected for fragmentation with 10 s dynamic exclusion time. Only ions with 2 and 3 charges were selected for MS/MS analysis. Secondary ions were isolated with a window of 1.6 *m/z*. For proteotyping isolates, peptides (0.15 μg) were desalted and then resolved as described here-above but using a 30 min gradient of mobile phase A and B. In this case, the gradient applied was: 2.5% B for 0 to 3 min, 2.5–25% B from 3 to 28 min, and 25–40% B from 28 to 33 min. The mass spectrometer acquisition was operated with the same settings as here above.

### MS/MS Data Interpretation

A database of 43,774 polypeptide sequences, representing the annotated genomes of *B. subtilis* subsp. *spizizenii* ATCC6633, *E. coli* BL21-Gold (DE3) pLysS AG, and *S. cerevisiae* S288C strain and totaling 15,766,031 amino acid residues, was used for assigning MS/MS spectra. For this interpretation, the Mascot Daemon software version 2.6.1 (Matrix Science) was set with 5 ppm peptide tolerance and 0.02 Da MS/MS fragment tolerance, 2 +and 3+ peptide charge, a maximum of two missed cleavages, carbamidomethylation of cysteine as fixed modification, oxidation of methionine as variable modification and trypsin as proteolytic enzyme. Mascot results were parsed with IRMa 1.31.1c software (Dupierris et al., 2009). The evaluation of isoelectric point (pI) and grand average of hydropathy (GRAVY) of identified peptides were obtained with Expasy Compute pI/MW^1^ and GRAVY calculator^2^, respectively. For proteotyping, MS/MS spectra analyzed were queried against the NCBInr database (01/03/2018 downloaded), comprising 108,307,546 protein sequences, using the Mascot daemon software set with 5 ppm peptide tolerance and 0.02 Da MS/MS fragment tolerance, 2+ and 3+ peptide charge, a maximum of one missed cleavage, carbamidomethylation of cysteine as fixed modification, oxidation of methionine as variable modification and trypsin as proteolytic enzyme. Peptide-to-Spectrum Matches were assigned with a *p* value below 0.05 in homology threshold mode. Proteins were validated when at least two different peptide sequences were observed. With these parameters, the false-positive rate was estimated to be below 1% for protein identification with the MASCOT decoy option search. The mass spectrometry proteomics data have been deposited to the ProteomeXchange Consortium via the PRIDE (Perez-Riverol et al., 2019) partner repository with the dataset identifier PXD014505 and 10.6019/PXD014505.

## Results

**FIGURE 1:**
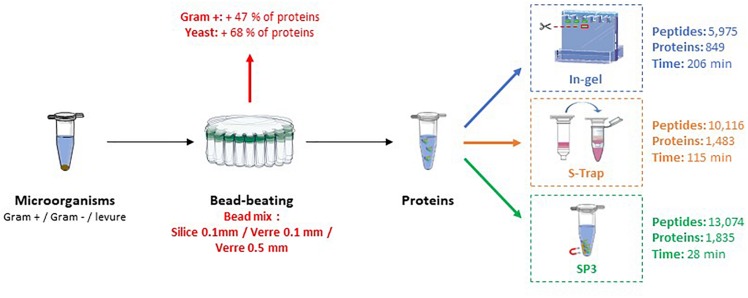
Overview of protein extraction and proteolysis comparison methods for a fast and performant sample preparation approach for proteotyping.

This study aims to propose a fast and efficient sample preparation for nanoLC-MS/MS proteotyping. The first part is focused on the lysis of microorganisms essentially done as described by Mappa et al. (2018) by the bead-beating approach after solubilization of cell pellets in presence of lithium dodecyl sulfate. The bead beating lysis was improved with adjustment of machine settings and bead types for greater protein extraction from both prokaryotic and eukaryotic organisms. In the second part, three protein digestion methods were optimized and then compared to establish the most efficient approach in terms of process time, and number of peptides and proteins identified. We also applied the selected method for proteotyping of several isolates to illustrate its applicability to medical samples. The measurement of protein yield by densitometry was performed with two replicates for each lysis condition, the comparison of digestion methods with three replicates for each condition and the application in pathogenic strains with one replicate per strain. Figure 1 shows an overview of the steps that have been optimized.

### Optimization of Bead-Beating Lysis for Fast Proteotyping by Tandem Mass Spectrometry

Because in-gel trypsin proteolysis delivers high quality peptide fractions ready for routine tandem mass spectrometry for microorganism identification, we first optimized a previous protocol by Mappa et al. (2018) based on mechanical disruption by bead-beating performed in a detergent-based lysis buffer (LDS) compatible with SDS-PAGE electrophoresis. The first stage of this protocol includes an incubation of 5 min at 99°C and a 5 min sonication in an ultrasonic water bath to dissolve aggregates. The second stage is the bead-beating treatment. We estimated the influence on protein extraction of three parameters: the strength of bead-beating agitation (3 or 10 cycles of 20 s, 3 or 10 cycles of 30 s, at maximum speed), the size and the nature of the beads (0.1 mm silica beads, 0.1 mm glass beads, 0.5 mm glass beads), and the effect of a mixture of these beads. The yield of protein extraction was estimated by densitometry measurement after SDS-PAGE electrophoresis and protein staining in comparison to a standard. For this, three microorganisms were used: *B. subtilis*, *E. coli*, and *S. cerevisiae* which have very differing cell envelopes and are models from Gram positive bacteria, Gram negative bacteria, and eukaryotic unicellular organisms, respectively.

**FIGURE 2:**
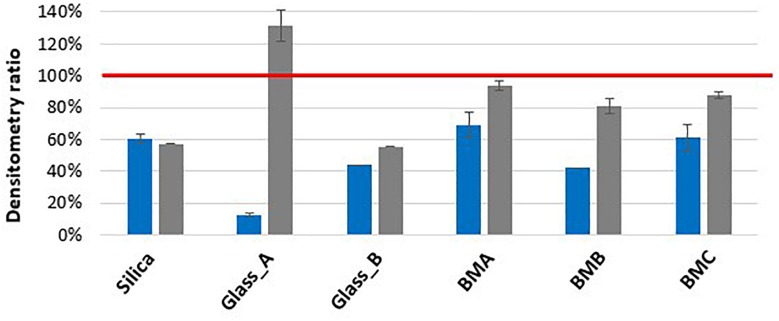
Protein extraction efficiency by bead beating for *B. subtilis* and *S. cerevisiae* samples compared to *E. coli.* Proteins obtained by bead beating (10 cycles of 30 s at 10,000 rpm) were subjected to denaturing electrophoresis and after Coomassie staining, samples were compared by densitometry (*n* = 2). The red line represents the *E. coli* densitometric reference, blue bar chart represents the *B. subtilis* densitometry ratio while gray one represents the *S. cerevisiae* densitometry ratio. Silica, silica beads 0.1 mm; Glass_A, glass beads 0.5 mm; Glass_B, glass beads 0.1 mm; BMA (Beads Mixture A), 2/3 silica beads 0.1 mm + 1/3 glass beads 0.5 mm; BMB (Beads Mixture B), 2/3 silica beads 0.1 mm + 1/3 glass beads 0.1 mm; BMC (Beads Mixture C), 1/3 silica beads 0.1 mm + 1/3 glass beads 0.5 mm + 1/3 glass beads 0.1 mm. Densitometry measurements are detailed on Supplementary Table S1.

In the condition where 0.1 mm silica beads were used with 3 rounds of 20 s and low speed agitation (7800 rpm), the lysis efficiency was found to be low for both Gram-positive bacteria and yeast with only 14 and 20% of protein extraction, respectively, compared to *E. coli*. The combination of a longer and more vigorous agitation (10 cycles of 30 s at 10,000 rpm) was shown to increase the protein extraction for these two microorganisms: fourfold increase for *B. subtilis* and threefold increase for *S. cerevisiae*. The bead types represented a second key parameter for a performant protein extraction (Figure 2 and Supplementary Table S1). The use of larger beads such as the 0.5 mm glass beads improved *S. cerevisiae* protein extraction (2.3 fold) in comparison to the 0.1 mm silica beads. However, the lysis with these beads decreased the protein extraction efficiency for *B. subtilis* by 4.8 fold. Smaller glass beads (0.1 mm) decreased *B. subtilis* protein extraction by 1.2 fold without impacting yeast lysis. With the objective to find the best compromise for simultaneous protein extraction from diverse microorganisms, we evaluated the protein extraction yield obtained with different bead mixtures that we named BMA, BMB, and BMC. As shown in Figure 2, BMA and BMC allowed the best extraction of proteins from the Gram-positive bacteria (densitometry estimates of 68 and 61%, respectively, compared to *E. coli* protein extraction), as well as from yeast (densitometry estimates of 93 and 88%, respectively). Both bead mixes gave similar lysis performances for *B. subtilis* in comparison to 0.1 mm silica beads but *S. cerevisiae* cell lysis is lower than when 0.5 mm glass beads are used alone. Replacement of 0.5 mm glass beads by 0.1 mm glass beads (BMB) is not efficient for protein extraction from *B. subtilis*. Lysis performances in the different conditions were measured by densitometry after protein denaturing electrophoresis and staining on duplicates. Using a single bead type is not adapted for an efficient protein extraction from prokaryotic and eukaryotic cells. In this case, we noted higher values of the standard deviation. In presence of a mixture of beads, the variability of lysis rate is lower (±10%).

**FIGURE 3:**
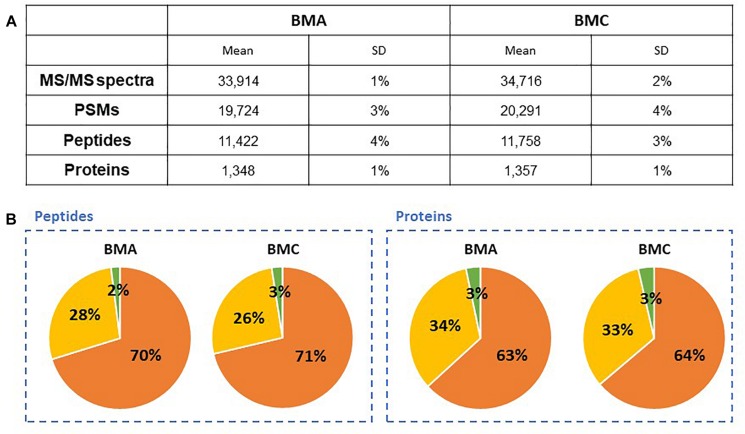
Proteomics-based comparison of mixtures of beads for cell lysis of Mix3. **(A)** NanoLC-MS/MS data for the Mix3 sample that includes *B. subtilis* (55%), *E. coli* (35%), and *S. cerevisiae* (10%) lysed either with BMA or BMC (*n* = 3). Only proteins validated with at least two peptides are considered (Supplementary Table S2). **(B)** Peptide and protein distributions amongst the three organisms present in the sample: *B. subtilis* (orange), *E. coli* (yellow) and *S. cerevisiae* (green).

To evaluate the bead-beating lysis efficiency of different microorganisms for a robust high-throughput procedure, an artificial mixture (Mix3) was assembled by using an asymmetric mixture in terms of number of cells of *B. subtilis* (55%), *E. coli* (35%), and *S. cerevisiae* (10%). The two bead mixtures BMA and BMC were used for testing the efficiency of protein extraction from Mix3, followed by SDS-PAGE electrophoresis, *in gel* digestion for 60 min and nanoLC-MS/MS. Figure 3A shows the results in terms of MS/MS spectra acquired, peptide assignation and protein identification. The identified proteins are listed in Supplementary Table S2. Both combinations of beads present similar lysis efficiency with rather limited variations of spectra (2%), peptides (3%) and unique peptides (3%), and a slightly higher number of proteins identified with at least two different peptides for the BMC condition (+9). The reproducibility of the mixture of beads is also observed from the proteomics results with less than ±5% of standard variation when comparing BMA and BMC performances with triplicates. In addition, proteins and peptides assigned to the three microorganisms do not differ significantly between both bead mixtures as shown in Figure 3B. The numbers of PSMs assigned to the organisms (75% for *B. subtilis*, 23% for *E. coli*, and 2% for *S. cerevisiae*) indicate protein biomass contributions reflecting loosely the initial ratio of cells. On this basis, we selected the bead mixture BMC as a greater bead mix for systematic use for MS/MS proteotyping. Of note, the global lysis efficiency of the whole procedure including the incubation at 99°C of the cells in presence of LDS, waterbath sonication, and bead-beating was estimated here. It would be interesting to quantify the effect of the different stages of the protocol and document to which extent microbial cells are lyzed before bead-beating treatment. For this, additional experiments with hard-to-lyze microorganisms, eventually in presence of matrices, used as controls could be of general interest.

### Faster Lysis and *In-Gel* Proteolysis

**FIGURE 4:**
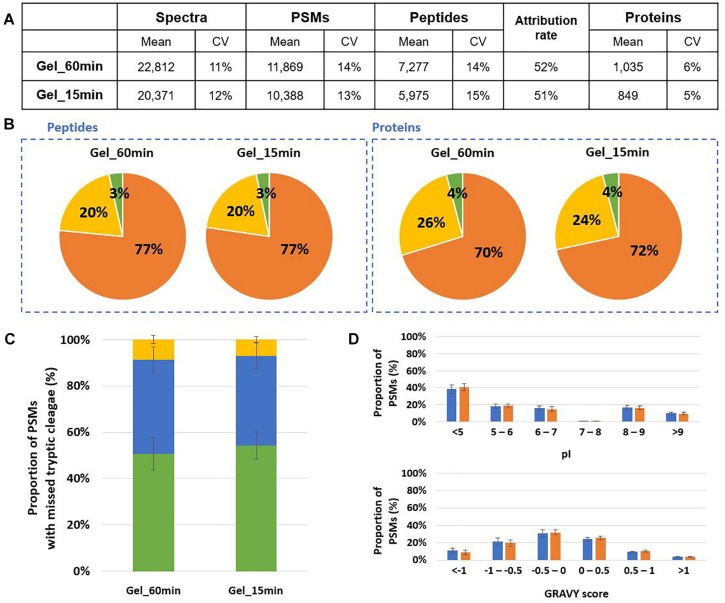
Comparison of 60 min and 15 min *in-ge*l proteolysis performances for Mix3. **(A)** NanoLC-MS/MS data for the triplicate analysis. Only proteins validated with at least two peptides are considered (Supplementary Table S2). **(B)** Peptide and protein distribution amongst the three organisms present in the sample: *B. subtilis* (orange), *E. coli* (yellow), and *S. cerevisiae* (green). **(C)** Proportion of peptides with 0 (green bars), 1 (blue bars) or 2 (yellow bars) missed cleavages. **(D)** Distribution of isoelectric point and hydrophobicity of peptides. The ratio of peptides obtained after 60 min and 15 min proteolysis are represented with blue and orange bars, respectively.

In order to shorten the time to perform the whole sample preparation before proteotyping we tested whether the lysis and in-gel proteolysis with trypsin could be simplified. The protein extraction was optimized, the previous bead-beating protocol including 2 boiling steps of 5 min and an ultrasonic bath of 5 min. We reduced the time of these steps at 2 min instead of 5 min. In this case, protein extraction increased 1.5 fold for *B. subtilis* and decreased 1.08 fold for *S. cerevisiae*. These adjustments did not impact significantly the lysis efficiency while the protocol could be reduced of 13 min. Then, we tested whether a digestion of 15 min instead of 60 min could be applied without diminishing the performances of proteotyping of Mix3. As shown in Figure 4, a total of 11,869 PSMs were assigned and 7,277 peptides were identified after a 60 min proteolysis corresponding to 1,035 proteins validated with at least 2 peptides (Figure 4A). When the time of digestion was reduced to 15 min, we assigned 88% of the PSMs previously obtained for the 60 min digestion, 82% of the peptides, and 82% of the proteins validated with at least 2 peptides. Enough peptides are obtained for the three microorganisms for their detection by proteotyping. The peptide and protein distributions were similar (Figure 4B). We further examined the effects of the digestion time on trypsin efficiency. As shown in Figure 4C, the same ratios of PSMs were observed for the 60 min and 15 min conditions independently of the number of missed cleavages. The physicochemical properties (pI and GRAVY index) of peptides from the two conditions present similar characteristics as shown in Figure 4D. Thus, no specific bias related to digestion time was observed on the type of peptides generated.

### On-Filter Proteolysis Enables a Faster Process Than In-Gel

**FIGURE 5:**
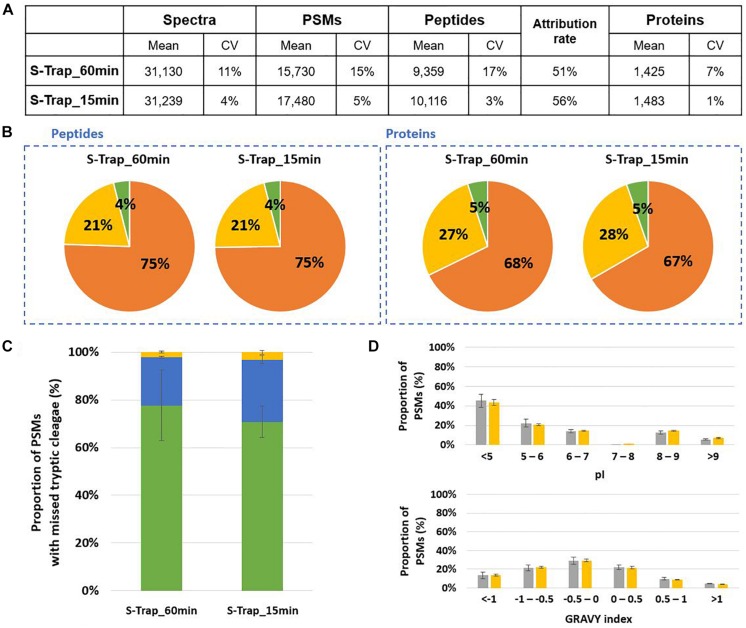
Comparison of 60 min and 15 min in-solution proteolysis of Mix3 using S-Trap protein purification mini-columns. **(A)** NanoLC-MS/MS data for the triplicate analysis. **(B)** Peptide and protein distribution amongst the three organisms present in the sample: *B. subtilis* (orange), *E. coli* (yellow) and *S. cerevisiae* (green). **(C)** Proportion of peptides with 0 (green bars), 1 (blue bars) or 2 (yellow bars) missed cleavages. **(D)** Distribution of isoelectric point and hydrophobicity of peptides. The ratio of peptides obtained after 60 min and 15 min proteolysis are represented with gray and yellow bars, respectively.

We evaluated the performance of S-Trap mini-columns for proteotyping using Mix3 as reference sample and performing the lysis in LDS buffer without the SERVA blue G250 and Phenol Red staining reagents, but supplemented with 5% beta-mercaptoethanol. Two duration of proteolysis, 15 and 60 min, were compared. Figure 5A shows that relatively similar results were obtained for both conditions. A slight increase in the number of peptides and proteins was observed for the shorter digestion. No differences were observed in terms of origin of the peptides and proteins as similar distribution ratios among the 3 model organisms were reported (Figure 5B). The trend of missed cleavages was not significantly modified but a slight increase was observed for the shorter proteolysis (Figure 5C). The pI and GRAVY index presented in Figure 5D were not discriminant parameters when comparing the peptides produced in both conditions. Noteworthy, the numbers of peptides and proteins identified by the S-Trap approach were similar amongst the replicates, but a better reproducibility between replicates was observed for short proteolysis.

### Rapidity and Simplicity of In-Solution Digestion Using Paramagnetic Beads

**FIGURE 6:**
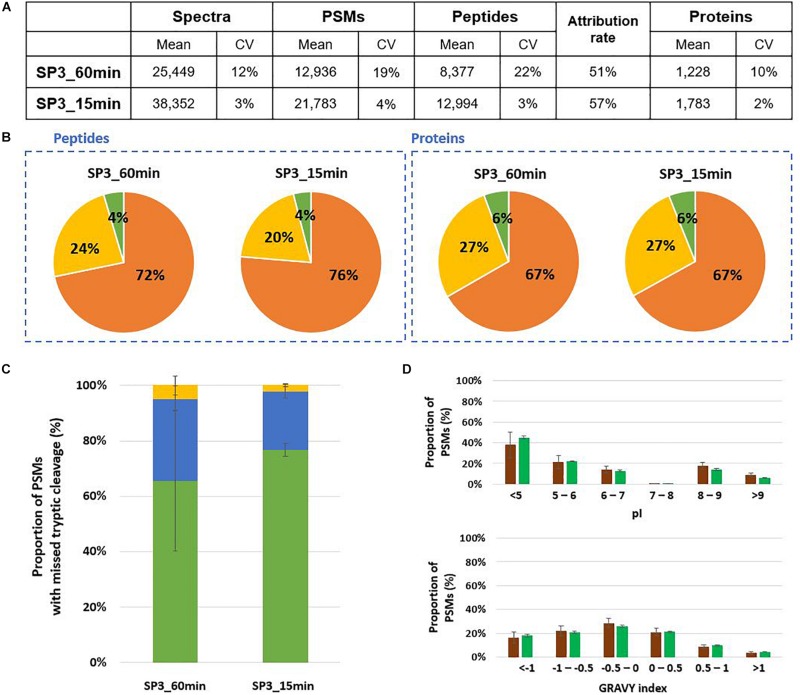
Comparison of 60 min and 15 min SP3 in-solution proteolysis of Mix3. **(A)** NanoLC-MS/MS data for the triplicate analysis. **(B)** Peptide and protein distribution amongst the three organisms present in the sample: *B. subtilis* (orange), *E. coli* (yellow), and *S. cerevisiae* (green). **(C)** Proportion of peptides with 0 (green bars), 1 (blue bars) or 2 (yellow bars) missed cleavages. **(D)** Distribution of isoelectric point and hydrophobicity of peptides. The ratio of peptides obtained after 60 min and 15 min proteolysis are represented with brown and green bars, respectively.

SP3 is another interesting alternative for sample preparation for tandem mass spectrometry proteotyping as this method by relying on paramagnetic beads opens the possibility of an automatized procedure. Here, the performances of the SP3 approach were evaluated using Mix3 as a reference sample under four different conditions: 60 min or 15 min proteolysis, 20 min or 2 min of protein binding, and with or without the reduction/alkylation step. For the lysis of microorganisms, the same protocol as for S-trap columns was applied, i.e., bead-beating in presence of LDS 1X and beta-mercaptoethanol. Compared to a 60 min proteolysis, the 15 min digestion allowed the identification of 55% more of peptides and 45% of additional proteins (Figure 6A). Peptides and proteins were attributed to the three organisms in comparable ratios (Figure 6B). The average number of tryptic missed cleavages was lower for the short proteolysis (Figure 6C). The physicochemical properties of peptides generated by both conditions were similar (Figure 6D). To shorten this protocol, we tested whether the protein binding of 20 min could be replaced by a 2 min binding, and also the application of the protocol without the reduction/alkylation step. No significant differences were observed in terms of peptides and proteins identified, tryptic missed cleavages and peptide properties (Supplementary Table S3). These adjustments enable to reduce the global sample preparation time by a factor of 1.7. In addition to increase significantly the identification of peptides and proteins, a short SP3 digestion presents a low variation between experimental triplicates whatever the condition.

### Compared Performances of the 3 Sample Preparation Methods

**FIGURE 7:**
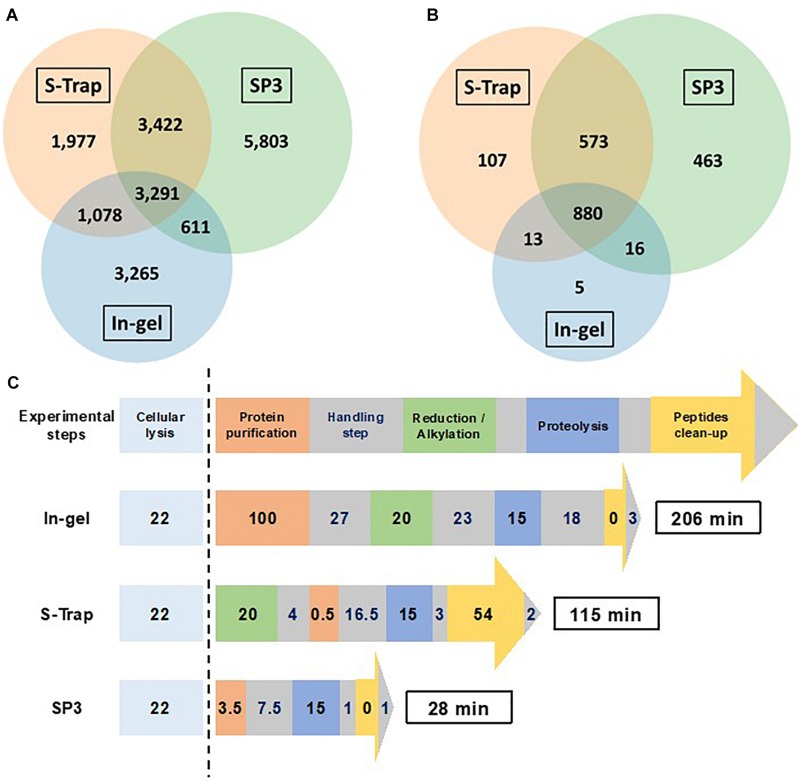
Comparison of in-gel (Gel_15 min), S-Trap (S-Trap_15 min) and SP3 (SP3_15 min) optimized protocols. **(A)** Venn diagram showing the overlap of peptide sequences identified for each sample preparation methods. **(B)** Venn diagram showing the overlap of proteins validated with at least two different peptides for each sample preparation methods. **(C)** Schematic representation of the workflow timing for the three sample preparation methods (numbers indicated the time in min required per step).

Figure 7 shows the performances of the three optimized workflows in terms of number of peptide sequences (A), number of identified proteins (B), and time necessary to perform the sample preparation (C). In-gel proteolysis in 15 min showed the weakest performances for these three criteria. The substitution of protein gel-trapping with filter purification lead to enhanced sample preparation yield with an increase in the number of peptide sequences of 19%. In-solution digestion with SP3 paramagnetic beads allowed an increase of peptide sequences identified of 59% in comparison to in-gel proteolysis. Interestingly, the peptides identified by the three approaches are not the same, and cumulating the three approaches allows identifying a total of 19,447 peptide sequences and 2,057 proteins. Specific peptides identified by only one of the three methods represented 57% of total peptide sequences, with 3,265 peptides for in-gel, 1,977 for S-Trap and 5,803 for SP3. Only 17% of the peptides are common to the three methods. SP3 shows the highest peptide coverage with 68% of the peptide pool. A total of 880 proteins (43%) are identified systematically by the three methods. The number of in-gel specific proteins are low (5 specific proteins) compared to S-Trap or SP3. Therefore, the three methods are highly complementary if the maximum of protein sequence coverage is seek, but SP3 only should be privileged if the objective is focused on protein identification.

Figure 7C shows the experimental time necessary to perform the three optimized protocols. In-gel digestion requires a total time of 206 min, including a long protein purification step of 127 min. Using S-Trap proteolysis the protocol can be performed in 155 min. SP3 optimized method without reduction/alkylation can be achieved in less than 30 min, with the enzymatic digestion step representing half of the protocol time.

### Application of the Optimized Lysis and SP3 Digestion Protocol for MS/MS Proteotyping of Bacterial Isolates

The optimized lysis and SP3 digestion protocol was applied to four pathogenic bacterial strains for their proteotyping by tandem mass spectrometry. The strains were *Bacillus cereus* (Sample 1), *Pseudomonas aeruginosa* (Sample 2), and *Acinetobacter baumannii* (Sample 3), as well as a clinical isolate from human urinary sample diagnosed as *Klebsiella aerogenes* (Sample 4). The four bacterial pellets were processed within 30 min and the resulting extracted peptides were analyzed by tandem mass spectrometry. MS/MS data were interpreted against the NCBInr generalist database and the identified peptide sequences were analyzed for ascertaining the microorganisms present in the sample. On this basis, the four bacteria could be recognized with a high degree of confidence as shown in Table 1. Even for *B. cereus* which does not encode many taxon-specific peptides at the species level because of its close phylogenetic proximity with numerous *Bacillus* species (Mesuere et al., 2016), a set of 15 of these were identified. A total of 5,607 PSMs could be assigned to *B. cereus* at the species level. For *K. aerogenes*, a total of 115 species-specific peptides were identified and 6,636 PSMs were assigned to this species. *P. aeruginosa* and *A. baumanii* are accurately identified at the species level with 7,384 assigned PSMs including 35 specific peptides for the former isolate and 8,521 PSMs with 202 specific peptides for the later isolate.

**TABLE 1:** Proteotyping by tandem mass spectrometry of four pathogenic bacteria.

**Sample**	**Species-specific peptides**	**Unique peptides (species level)**	**PSMs (species level)**	**Identified species**
1	15	3,918	5,607	*Bacillus cereus*
2	35	5,508	7,384	*Pseudomonas aeruginosa*
3	202	6,334	8,521	*Acinetobacter baumannii*
4	115	4,460	6,636	*Klebsiella aerogenes*

## Discussion

In this study we optimized the sample preparation of microorganisms prior their identification by proteotyping with tandem mass spectrometry. For this, we evaluated the yield of protein extraction from a mixture of three representative microorganisms, including a Gram-negative bacterium, a Gram-positive bacterium and a yeast. Gram-positive bacteria and yeasts are known to be more resistant to lysis than Gram-negative bacteria. This difference may lead to strong biases in estimating biomass contributions if the extraction of proteins from complex samples is not optimized. The protocols presented here are based on mechanical disruption of cells with bead-beating which has been proved of high value for difficult-to-lyse microorganisms compared to protocol relying only on sonication (Lanigan et al., 2004) and should be amenable to automation. Beside a comparison of three protein extraction methods, we also optimized the bead-beating cell disruption which is already described as a powerful cellular lysis approach. We adapted the mixture of beads with variable size, structure, and composition and the settings in order to obtain the best results for a large diversity of microorganisms. Our results indicate that the use of harsh conditions, i.e., vigorous and long disruption enabled a significant improvement of protein extraction for *B. subtilis* and *S. cerevisiae*. The size of the beads used for bead-beating resulted of crucial importance. Indeed, different sizes are recommended depending on the microorganisms to lyse as bacterial cells which are between 0.2 and 2 μm in size are much smaller than eukaryotic cells. As previously reported, better lysis performances were obtained for *S. cerevisiae* using 0,5 mm glass beads (Sasidharan et al., 2012) and for *B. subtilis* using 0,1 mm silica beads (Swarge et al., 2018). Our proposal for a mixture of beads represents a very good compromise for prokaryotic and eukaryotic cell disruption using the same protocol and same lysis tube. The results obtained with two mixtures of silica and glass beads, BMA and BMC, were relatively comparable. We recommend the use of BMC which comprises silica beads (0.1 mm) and glass beads of two sizes (0.1 and 0.5 mm) for proteotyping a large diversity of prokaryotic and eukaryotic microorganisms. In-gel, S-Trap and SP3 proteolysis methods were compared for fast delivery of peptides for proteotyping. Using in-gel with a short digestion step of 15 min decreases the number of peptides and proteins identified compared to a protocol including a 60 min proteolysis but does not impact the proportion of missed cleavages. In opposition to in-gel method, S-Trap and SP3 digestions are faster and slightly improve the peptide yields, as well as their quality with a larger proportion of peptides without tryptic missed cleavage and without any notable change in terms of physicochemical properties.

S-Trap proteolysis is based on protein retention onto a quartz filter to avoid matrix interference during digestion and the use of a hydrophilic filter to adsorb enzyme solution and increase the enzyme contact with proteins. S-Trap proteolysis is easy to implement, but requires multiple centrifugation steps. Several studies have already presented the performances of S-Trap for efficient removal of detergents (Elinger et al., 2019) and improved protein digestion (Ludwig et al., 2018) for example. Here, we have shown that these mini-columns are compatible with extracts obtained with bead-beating in presence of LDS 1X and the digestion can be shortened by using higher amount of trypsin. S-Trap proteolysis represents a valuable alternative method for solid phase sample preparation and allows a fast purification and digestion. Use of S-Trap for complex sample has been described with sputum (HaileMariam et al., 2018) and saliva (Lin et al., 2019). The required amounts of starting material were 50 μg and 5 mL, respectively.

Our results show that the protocol based on SP3 paramagnetic beads outperforms the two other approaches in terms of speed and yield of extracted peptides. The SP3 in-solution digestion is efficient even for short proteolysis. Surprisingly, longer proteolysis leads to a loss of peptides, which can be explained by several factors including the binding or non-specific adsorption of peptides on beads, their adsorption on tube walls or peptide aggregation (Zheng and DeMarco, 2017). To further shorten the global process several changes were introduced to the previously published protocol. As proposed by Moggridge et al. (2018), the step of protein fixation to paramagnetic beads can be reduced to 2 min, without any significant impact. Here, lysis was performed using LDS 1X supplemented with beta-mercaptoethanol for unfolding proteins and maintaining them as this until fixation to the beads. The reduction/alkylation step was omitted without major impact on the digest quality as shown by the mean ratio of missed cleavages. These improvements enable a drastic reduction of the SP3 protocol, with an operation time decreased from 120 min to only 30 min. Therefore, the optimized SP3 protocol outperforms the two other methods tested in this work. Previous works have also highlighted the potential of SP3 method compared to other approaches, such as FASP and InStageTip (Sielaff et al., 2017), but an excess of proteins may affect its performance as recently shown (Hughes et al., 2019). SP3 has been applied on a large number of complex samples such as human bones (Cleland, 2018). The material used was really low, i.e., 2.5 mg, and required less than a microgram of proteins in most cases (Hughes et al., 2019).

## Conclusion

In conclusion, we proposed to lyse the cells from a complex sample that may contain very diverse microorganisms by bead-beating using drastic agitation conditions with a mixture of silica and glass beads of different sizes. In-gel, on-filter (S-Trap) and in-solution (SP3) proteolysis of extracted proteins were optimized to reduce the digestion step to 15 min. On the Mix3 sample, we could identify 8,245, 9,768, and 13,127 unique peptides by in-gel, S-Trap, and SP3 protocols, respectively. Thus, SP3 outperforms the two other protocols in terms of speed and yield of peptides. Interestingly, the three methods increase considerably the peptidome coverage when merged with the detection of a total of 19,447 peptide sequences (3,265, 1,977, and 5,803 unique peptides by in-gel, S-Trap, and SP3 protocols, respectively). The performances, cost, adaptability of the SP3 protocol to a 96-well microplate format, and the possibility of automation make it as the method of choice in the sample preparation workflow for high-throughput MS/MS-based proteotyping of microorganisms. Evaluation of the robustness and gains of such optimized protocol in the clinical settings is now a valuable step to be done in the near future for offering tandem mass spectrometry-based proteotyping as a diagnostic tool in clinical microbiology.

## Data Availability

The datasets generated for this study can be found in the mass spectrometry proteomics data which have been deposited to the ProteomeXchange Consortium via the PRIDE (Perez-Riverol et al., 2019) partner repository with the dataset identifier PXD014505 and 10.6019/PXD014505.

## Author Contributions

KH, JA, and BA-B conceived the study and drafted the manuscript with the help from all other co-authors. KH, DG, and BA-B performed the experimental work with the assistance of LG, OP, and JA. KH, DG, LG, OP, JA, and BA-B analyzed the data.

## Conflict of Interest Statement

The authors declare that the research was conducted in the absence of any commercial or financial relationships that could be construed as a potential conflict of interest.

## Footnotes

1 http://web.expasy.org/compute_pi/

2 http://gravy-calculator.de/
